# Classification of Mycena and *Marasmius* Species Using Deep Learning Models: An Ecological and Taxonomic Approach

**DOI:** 10.3390/s25061642

**Published:** 2025-03-07

**Authors:** Fatih Ekinci, Guney Ugurlu, Giray Sercan Ozcan, Koray Acici, Tunc Asuroglu, Eda Kumru, Mehmet Serdar Guzel, Ilgaz Akata

**Affiliations:** 1 Institute of Artificial Intelligence, Ankara University, Ankara 06100, Türkiye; fatihekinci@ankara.edu.tr (F.E.); kacici@ankara.edu.tr (K.A.); mguzel@ankara.edu.tr (M.S.G.); 2 Department of Computer Engineering, Faculty of Engineering, Başkent University, Ankara 06790, Türkiye; guneyugurlu@baskent.edu.tr (G.U.); gozcan@baskent.edu.tr (G.S.O.); 3 Department of Artificial Intelligence and Data Engineering, Faculty of Engineering, Ankara University, Ankara 06830, Türkiye; 4 Faculty of Medicine and Health Technology, Tampere University, 33720 Tampere, Finland; 5 VTT Technical Research Centre of Finland, 33101 Tampere, Finland; 6 Graduate School of Natural and Applied Sciences, Ankara University, Ankara 06830, Türkiye; ekumru@ankara.edu.tr; 7 Department of Computer Engineering, Faculty of Engineering, Ankara University, Ankara 06830, Türkiye; 8 Department of Biology, Faculty of Science, Ankara University, Ankara 06100, Türkiye; akata@science.ankara.edu.tr

**Keywords:** macrofungi classification, machine learning, deep learning, self-organizing maps, MaxViT-Small

## Abstract

Fungi play a critical role in ecosystems, contributing to biodiversity and providing economic and biotechnological value. In this study, we developed a novel deep learning-based framework for the classification of seven macrofungi species from the genera *Mycena* and *Marasmius*, leveraging their unique ecological and morphological characteristics. The proposed approach integrates a custom convolutional neural network (CNN) with a self-organizing map (SOM) adapted for supervised learning and a Kolmogorov–Arnold Network (KAN) layer to enhance classification performance. The experimental results demonstrate significant improvements in classification metrics when using the CNN-SOM and CNN-KAN architectures. Additionally, advanced pretrained models such as MaxViT-S and ResNetV2-50 achieved high accuracy rates, with MaxViT-S achieving 98.9% accuracy. Statistical analyses using the chi-square test confirmed the reliability of the results, emphasizing the importance of validating evaluation metrics statistically. This research represents the first application of SOM in fungal classification and highlights the potential of deep learning in advancing fungal taxonomy. Future work will focus on optimizing the KAN architecture and expanding the dataset to include more fungal classes, further enhancing classification accuracy and ecological understanding.

## 1. Introduction

Fungi are fundamental components of ecosystems, contributing significantly to various sectors, including healthcare, agriculture, and environmental restoration. The Basidiomycota division, one of the most diverse groups within the fungal kingdom, comprises over 41,000 species globally [1]. Agaricales is particularly notable among its orders, encompassing approximately 20,000 species spread across 509 genera and 46 families [2]. Within this order, genera such as *Mycena* and *Marasmius* exhibit remarkable ecological diversity and morphological adaptation, making them essential for biodiversity studies [2,3].

The genus *Mycena* is characterized by saprotrophic species with slender, fragile stripes and striate caps that vary in shape and color, ranging from subdued tones of gray and brown to vibrant hues like yellow, red, and blue [4]. Several species within this genus, including *Mycena crocata*, *Mycena epipterygia*, and *Mycena pura*, have been studied for their unique morphological traits and ecological preferences [5,6,7]. Similarly, *Mycena seynii* thrives in coastal regions, growing on decaying Pinus cones and displaying distinct cap striations and vinaceous stipes [8].

In contrast, the genus *Marasmius* includes small- to medium-sized fungi with a wide range of ecological roles, from saprotrophy to potential mycorrhizal associations [3,9]. Species such as *Marasmius* oreades and *Marasmius* rotula are widely recognized for their ecological and culinary importance, the former forming iconic fairy rings in temperate regions and the latter displaying unique collariate lamellae [3,7].

Artificial intelligence (AI) advancements, particularly in deep learning (DL), are transforming fungal taxonomy and biodiversity research [10]. Distinguishing morphologically similar fungi species in images remains a key challenge, compounded by the need for large, well-annotated datasets to ensure that deep learning models generalize effectively. Traditional fungal classification methods further exacerbate these challenges, requiring expert knowledge, time-consuming processes, and struggling to handle large datasets. While deep learning models offer advantages in automation and accuracy, they also face limitations such as data scarcity, class imbalance, and generalization issues, which must be addressed for practical implementation. The existing methods attempt to tackle these issues: CNN-based models improve classification accuracy but lack interpretability, while pretrained networks like ResNet and EfficientNet achieve strong performance but may not generalize well to ecological datasets. Unsupervised clustering techniques such as self-organizing maps (SOMs) offer improved feature representation but are rarely applied in fungal classification. Our work bridges this gap by proposing a CNN-SOM model that enhances both accuracy and interpretability. Specifically, we introduce a novel approach to leveraging SOM in a supervised setting, providing a more robust and interpretable solution for fungal classification. Traditional fungal identification methods often require specialized expertise and significant time investment, limiting their scalability for large datasets [11]. In contrast, DL models provide rapid and precise identification by analyzing complex morphological features, thereby overcoming the limitations of conventional approaches [12]. These technologies enhance taxonomic accuracy and facilitate public engagement through accessible mobile applications, encouraging broader participation in biodiversity initiatives [13]. Furthermore, integrating environmental data into DL frameworks supports ecological research and conservation efforts by improving the understanding of fungal diversity and distribution [14].

The classification of fungi, particularly within the *Mycena* and *Marasmius* genera, is not solely a computational task but an integral part of ecological and taxonomic studies. Ecologically, these fungi play crucial roles in nutrient cycling and ecosystem stability. Their correct identification helps us understand their ecological interactions, distribution patterns, and potential applications in conservation efforts. Taxonomically, accurate classification supports systematic studies, species delimitation, and biodiversity assessments. Traditional classification methods rely on expert knowledge and manual observations, which are time-consuming and error-prone. By integrating deep learning with an ecological and taxonomic approach, we provide a scalable and efficient tool for fungal identification, aiding researchers, conservationists, and citizen scientists in biodiversity monitoring and taxonomy.

This study leverages DL to develop a robust classification model for seven macrofungi species: *Mycena crocata*, *Mycena epipterygia*, *Mycena pura*, *Mycena rosea*, *Mycena seynii*, *Marasmius oreades*, and *Marasmius rotula*. These species were selected based on their ecological importance and distinct morphological characteristics. The proposed DL model aims to provide accurate, efficient identification, contributing to scientific research and promoting the public awareness of macrofungal biodiversity while supporting conservation and sustainable utilization efforts. In this study, feature extraction was performed on an image dataset using a convolutional neural network (CNN) model, and the extracted features were classified using a Kohonen self-organizing map (SOM). The aim of the study is to analyze the performance of traditional CNN models and SOM on complex datasets and to evaluate the applicability of these techniques. To the best of our knowledge, this study is the first to utilize the SOM, typically employed in unsupervised learning, in conjunction with a CNN for the macrofungi classification problem. The main contribution of the manuscript is presenting a novel methodology that demonstrates the effectiveness of deep learning models in the classification of macrofungal species belonging to the *Mycena* and *Marasmius* genera. Specifically, this study is innovative in applying Kohonen self-organizing maps (SOMs) and Kolmogorov–Arnold Network (KAN) layers for fungal taxonomy for the first time. Furthermore, it is one of the first studies to demonstrate the superior performance of cutting-edge deep learning architectures, such as MaxViT-S, in fungal classification.

The rest of this paper is structured as follows: In the Section 2, the dataset utilized in the study is analyzed, and the definitions of the pretrained models and experimental setup are given. In the Section 3, evaluation metrics and empirical results are explained. The article concludes with the Section 4, where the limitations of the study and potential directions for future research are addressed.

## 2. Materials and Methods

### 2.1. General Framework

**Figure 1:**
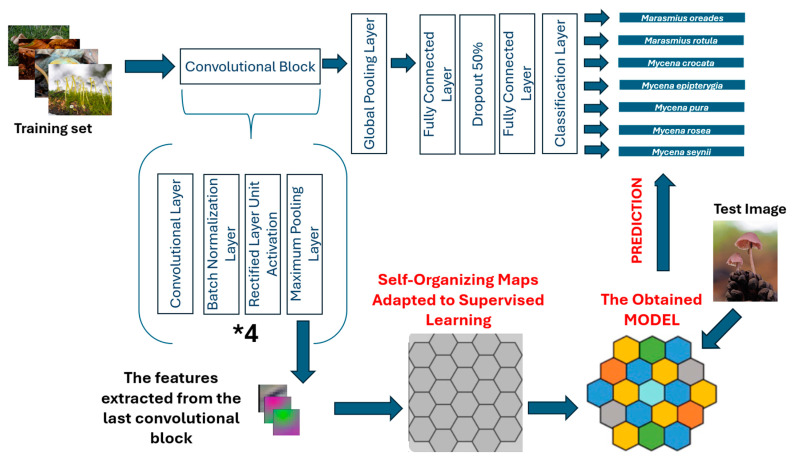
The proposed CNN-SOM architecture.

In this study, our aim is to predict the class (species) of a macrofungus. Initially, a CNN architecture was proposed and subsequently utilized for feature extraction. The features extracted from the proposed CNN architecture were classified using a SOM architecture adapted for supervised learning. The general framework for the proposed method can be seen in Figure 1.

A custom-designed CNN architecture extracts high-dimensional feature representations from fungal images. The output of the last convolutional block serves as the feature vector. SOM is used to map high-dimensional features into a structured, interpretable 2D representation, preserving topological relationships between features. Unlike traditional classifiers, SOM enables the visual clustering of similar species. The SOM network assigns each feature vector to the most similar neuron, determining the fungal species.

The SOM network was chosen for its capability to handle high-dimensional data, cluster similar features, and preserve the topological relationships of feature spaces. Unlike conventional classifiers, SOM provides a structured visualization of feature distributions, helping to interpret the classification process.

- Discriminative Features Used: The classification relied on the deep features extracted using CNNs. These features include the following:
  - Texture and Surface Patterns: Identifying fine-grained details like cap striations, surface roughness, and gill structures.
  - Color Variations: Differentiating species based on pigmentation variations influenced by age and environmental conditions.
  - Morphological Shapes: Capturing the unique structural attributes of fungi, such as cap, stipe, and gill arrangements.
  - Deep Representations: Extracted from CNN layers, enabling the model to learn hierarchical feature relationships that distinguish species.

Using SOM in combination with CNN improves classification accuracy by refining feature space clustering, making it particularly effective for species with subtle morphological differences.

### 2.2. Dataset

**Figure 2:**
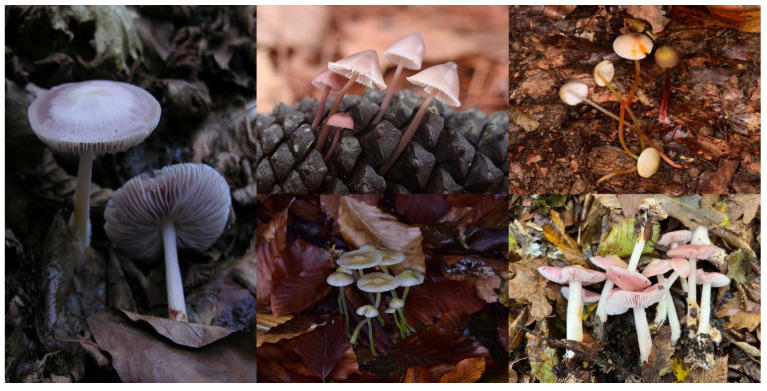
A macroscopic overview of *Mycena* species.

**Figure 3:**
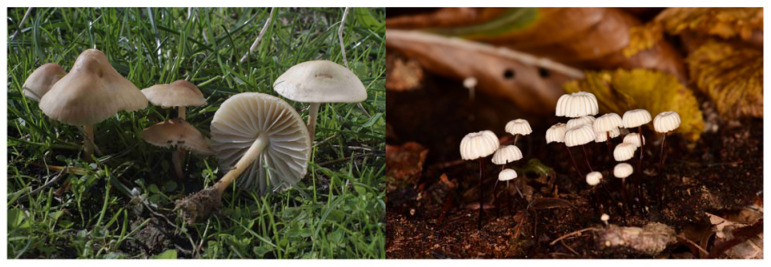
A macroscopic overview of *Marasmius* species.

The dataset utilized in this study was collected from Global Core Biodata Resource (GCBR) [15]. This dataset can be accessed in [16]. In the dataset, there are 7 classes of fungi. A total of 5 classes belong to *Mycena* (*Mycena, pura*, *Mycena seynii*, *Mycena crocata*, *Mycena epipterygia*, and *Mycena rosea*) and 2 classes belong to *Marasmius* (Marasmius oreades and *Marasmius rotula*). Macroscopic overviews of *Mycena* and *Marasmius* are demonstrated in Figure 2 and Figure 3, respectively.

According to Figure 2, the first column represents *Mycena pura*, the second column represents *Mycena seynii* and *Mycena epipterygia* from top to down, respectively, and the third column represents *Mycena crocata* and *Mycena rosea* from top to down, respectively.

According to Figure 3, the first column and the second column represent *Marasmius oreades* and *Marasmius rotula*, respectively.

In the collected dataset, there are 1582 samples that belong to 7 mushroom species. As can be seen from Table 1, there are 222, 228, 229, 243, 220, 227, and 213 samples for *Marasmius oreades*, *Marasmius rotula*, *Mycena crocata*, *Mycena epipterygia*, *Mycena pura*, *Mycena rosea*, and *Mycena seynii*, respectively.

The dataset was split into a training set and an independent test set. The training set comprises 70% of the dataset, whereas the independent test set includes the remaining 30%. Table 2 shows the distribution of the samples according to the training and independent test sets.

### 2.3. Methods

A self-organizing map (SOM) is an unsupervised learning method used for dimensionality reduction and clustering [17]. It projects high-dimensional data onto a low-dimensional grid while preserving topological structure. Unlike traditional neural networks, SOMs rely on competitive learning with a neighborhood function. They are widely applied in domains such as remote sensing and biosignal processing. The key advantage of SOMs lies in their ability to simplify complex data visualization and reveal underlying patterns, making them valuable for data-driven modeling [18,19,20].

The Kolmogorov–Arnold representation theorem in Kolmogorov–Arnold Networks (KANs) offers a promising research direction in neural networks [21]. KAN enhances efficiency and enables more effective solutions in domains like time series forecasting and remote sensing [22,23]. As an advanced alternative to multilayer perceptron (MLP) models, KAN differs by making activation functions learnable rather than fixed [24]. This is achieved through parameterizable spline functions integrated into the network [25]. The theorem states that any continuous multivariate function can be represented as a composition of univariate functions, enhancing the model’s ability to capture complex non-linear relationships [26].

GoogleNet, or Inception v1, is a deep convolutional neural network introduced by Szegedy et al. [27] in 2014. Designed for the ImageNet Large Scale Visual Recognition Challenge (ILSVRC), it achieved state-of-the-art performance with its novel “Inception Module,” which integrates convolutional filters of different sizes to enhance multi-scale feature extraction [28]. GoogleNet emphasizes computational efficiency through dimensionality reduction and sparse connections. A key innovation is the Inception module, which combines convolutions of various filter sizes (1 × 1, 3 × 3, and 5 × 5) with max pooling to capture diverse features while reducing computational costs [27]. To address the vanishing gradient problem, auxiliary classifiers provide gradient signals to earlier layers, improving convergence. Instead of traditional fully connected layers, GoogleNet employs global average pooling to reduce overfitting and parameter count. These optimizations enable deep feature learning while maintaining efficiency [28].

VGG19 is a deep convolutional neural network introduced by Simonyan and Zisserman in 2014 as a part of the Visual Geometry Group’s contribution to ILSVRC [29]. It features a simple and uniform design, using small 3 × 3 convolutional filters throughout the architecture, making it a benchmark for image recognition tasks [30]. The network consists of 19 weight layers—16 convolutional and 3 fully connected—organized into five convolutional blocks, each followed by max pooling for spatial reduction [29]. ReLU activation functions are applied after each layer to introduce non-linearity. The final fully connected layers culminate in a softmax classifier for prediction. This structured approach ensures effective feature extraction while maintaining computational efficiency [30].

MobileNetV3 is a lightweight and efficient convolutional neural network designed for mobile and embedded vision applications [31]. It builds on MobileNetV2 by using inverted residual blocks with linear bottlenecks, which reduce computational cost while preserving critical information [10]. Optimized through neural architecture search, MobileNetV3 fine-tunes layer configurations for latency and accuracy across different hardware platforms. It incorporates Squeeze-and-Excitation (SE) modules to enhance important feature responses and uses the Hard-Swish activation function for improved efficiency [31]. The architecture has two variants: MobileNetV3-Large for higher accuracy and MobileNetV3-Small for low-resource environments [31]. A global average pooling layer minimizes parameters before the final classification stage, ensuring efficiency while maintaining accuracy [10].

ResNetV2 is an improved version of the original Residual Network (ResNet), designed for better training stability and accuracy in deep neural networks [32]. It modifies the residual block structure by applying batch normalization and ReLU activation before convolutions, which stabilizes training and enhances convergence [33]. ResNetV2 employs identity skip connections to improve gradient flow, mitigating the vanishing gradient problem in very deep networks. It uses a bottleneck block structure with 1 × 1 and 3 × 3 convolutions to balance computational efficiency and representational power. The architecture is available in multiple variants, including ResNet50V2, ResNet101V2, and ResNet152V2, with the number indicating total layers [32].

EfficientNet-B0, introduced by Tan and Le in 2019, is a highly efficient baseline model that achieves high accuracy with fewer parameters and FLOPs compared to other CNNs [34]. It utilizes a compound scaling method to optimize network depth, width, and input resolution, balancing model size, accuracy, and efficiency [35]. The core building block is the Mobile Inverted Bottleneck Convolution (MBConv) layer, which includes depthwise separable convolutions, linear bottlenecks, and SE blocks to reduce computational cost and improve feature representation [35]. EfficientNet-B0 is organized into several stages of MBConv layers, followed by global average pooling and a fully connected classification layer. The Swish activation function is used to improve gradient flow and performance in deeper layers, while global average pooling reduces the parameter count [34].

EfficientNetV2, introduced by Tan and Le in 2021, improves upon EfficientNet with faster training, better scalability, and higher accuracy [36]. It refines the compound scaling approach by dynamically adjusting depth, width, and resolution for faster training, particularly on modern hardware [37]. A key innovation is the fused MBConv block, which replaces depthwise separable convolutions with standard convolutions for faster processing in early network stages. In later stages, MBConv layers from EfficientNetB0 are used for efficiency [36]. The model employs regularization techniques like stochastic depth, randaugment, and mixup to enhance generalization and speed up training [36]. EfficientNetV2 offers variants optimized for different dataset sizes: EfficientNetV2-Small for small datasets, EfficientNetV2-Medium for balanced performance, and EfficientNetV2-Large for large datasets requiring high accuracy [37]. It uses Swish activation for gradient flow and ReLU6 in the fused layers for efficiency.

Maximized Vision Transformer (MaxViT) is a hybrid model combining CNNs and multi-axis attention for efficient computation and high performance [38]. MaxViT-Small is a lightweight version designed for resource-constrained environments [39]. It processes images hierarchically, using local (window) and global (grid) attention mechanisms for efficient multi-scale feature extraction. The model includes feed-forward networks (FFNs) for feature interaction and a convolutional stem for initial feature extraction [40]. MaxViT-Small avoids explicit positional embeddings by using attention mechanisms. It ends with global average pooling and fully connected layers for classification, using GELU activation for enhanced training stability. The model achieves competitive accuracy with low computational cost [41].

### 2.4. Experimental Setup

The pretrained models selected as baselines for our study and the rationale behind their selection are explained below:

GoogleNet was selected since it has demonstrated exceptional performance on large-scale image classification tasks and achieved state-of-the-art results. MobileNet-V3-Large is a lightweight model and optimized for resource-constrained settings, which aligns with our requirements. ResNet-V2-50 has strong feature extraction capabilities for diverse datasets including our small-sized dataset. EfficientNet-B0 offers a balance between accuracy and computational efficiency, making it suitable for small datasets with limited GPU memory. It was selected since larger EfficientNet models do not fit within our GPU memory constraints. EfficientNet-V2-M provides enhanced performance over the earlier versions. VGG19 is a simpler and deeper network with robust feature extraction capabilities, often working well for small datasets despite higher computational demands. MaxVit-S combines convolutional and attention mechanisms, enabling it to capture both local and global features effectively.

For the pretrained models, three hyperparameters were optimized by utilizing a random search approach to identify the optimal configuration in all the models. These are batch size [16, 32, 64], learning rate [1 × 10^−2^, 1 × 10^−3^, 1 × 10^−4^], and weight decay [1 × 10^−3^, 1 × 10^−4^, 1 × 10^−5^]. Random search is a hyperparameter optimization technique that involves randomly sampling values from a predefined range for each hyperparameter and evaluating their performance. Unlike grid search, which exhaustively explores all combinations of hyperparameter values, random search selects a subset of combinations based on randomness, offering a more computationally efficient alternative, particularly in high-dimensional search spaces. This method is advantageous when certain hyperparameters have minimal impact on the model’s performance, allowing it to focus on identifying promising regions of the search space without testing every possible combination.

The Adam optimizer and the step learning rate (StepLR) scheduler were utilized in the training process. The StepLR scheduler reduces the learning rate of each parameter group by a specified factor at fixed intervals. In our case, we applied a decay rate of 0.1 every 7 epochs. The StepLR scheduler helps the model converge more effectively by allowing it to take larger steps initially and then progressively smaller steps as training progresses. For GoogleNet and MobileNet-V3-L, the training process converged after 10 epochs, whereas for the other pretrained models it required 20 epochs to complete. In addition, to reduce overfitting, the images were shuffled at the beginning of each epoch.

To augment the training set, PyTorch (v2.6)’s Random Horizontal Flip, random rotation, and color jitter techniques were applied. For the random rotation method, the rotation range was set between −15 and 15 degrees. The brightness, contrast, saturation, and hue parameters for the color jitter method were set to 0.1, 0.1, 0.1, and 0.05, respectively. These augmentations generate an infinite variety of augmented images. The number of augmentations is dynamic and depends on the number of epochs and the frequency with which each sample is loaded. Although the transformations do not explicitly expand the dataset size, they effectively enable the model to encounter multiple variations in the same data, thereby enhancing its generalization capabilities.

The CNN model utilized in the study was built and enhanced upon a classical custom CNN architecture. The model consists of the following components:

- A total of 4 convolutional blocks, and each has 2D convolutional, batch normalization, ReLU activation, and maximum pooling layers.
- Global Average Pooling (GAP): The feature maps generated by the convolutional blocks are summarized using Adaptive Average Pooling. This technique helps prevent overfitting by reducing the number of parameters.
- Fully Connected Layers: The first fully connected layer consists of 512 neurons and incorporates dropout with a rate of 0.5. The second fully connected layer contains 256 neurons. The final layer is a linear layer with 7 neurons, corresponding to the number of classes.

The developed CNN architecture is presented in Table 3.

All the images were resized to dimensions of 224 × 224 for the proposed CNN architecture. During the training phase, the same augmentation methods as those used for the pretrained models were applied. Similarly, as with the pretrained models, the Adam optimizer and the StepLR scheduler were employed to optimize the hyperparameters, learning rate, weight decay, and batch size utilizing a random search approach. Due to early stopping, the training process converged after 33 epochs. The shuffling mechanism was also applied to reduce overfitting.

In this study, SOM, an unsupervised neural network, was adapted for our fungi classification task. Features were extracted from the final convolutional block layer of the proposed CNN model. These features were analyzed using the SOM model implemented with the MiniSom library [42]. In the proposed CNN, the output of the 4th convolutional block has dimensions of 14 × 14 × 256, resulting in each sample provided to the SOM containing 50,176 features. The parameters optimized during the training phase were grid size, sigma, learning rate, and the number of iterations, with respective values of 20 × 20, 1.0, 0.5, and 5000.

The proposed CNN-SOM model consists of two main components:

1. Feature Extraction Using a CNN
  - A custom CNN architecture is employed to extract high-dimensional features from fungal images.
  - The CNN consists of four convolutional blocks, each comprising a convolutional layer, batch normalization, ReLU activation, and max pooling to progressively reduce spatial dimensions while retaining crucial feature information.
  - The output of the CNN is a high-dimensional feature vector (14 × 14 × 256 = 50,176 features).
2. Feature Clustering and Classification Using SOM
  - The self-organizing map (SOM) is a type of neural network used for dimensionality reduction and clustering.
  - The extracted CNN features are mapped into a 2D topological grid, where similar fungal species are grouped together.
  - Unlike traditional classifiers, SOM provides a structured and interpretable representation of species similarities.
  - A Best Matching Unit (BMU) approach is used to classify new images based on the closest neuron in the SOM grid.

The CNN extracts visual features, such as texture, shape, and color variations in fungal species. The final layer’s output is not directly classified; instead, the feature vectors are fed into SOM for clustering and classification. Once trained, SOM classifies new samples using the following steps:

1. Extract Features from CNN: A test image is passed through CNN to generate a feature vector.
2. Find the Best Matching Unit (BMU): The neuron in the SOM grid closest to the input feature vector is identified.
3. Assign a Class Label: Each neuron is assigned a fungal species label based on the majority class of training samples mapped to it.
4. Final Prediction: The class of the BMU is assigned to the test image.

This process ensures that the model learns complex feature distributions while maintaining interpretability.

Traditional CNN models rely on a fully connected classifier at the final stage. However, our approach replaces this with SOM, providing several advantages such as improved interpretability, better feature clustering, and generalization to new samples. Unlike dense classifiers, SOM visualizes how species relate to each other. SOM preserves topological relationships, ensuring similar species are grouped correctly. SOM is less prone to overfitting compared to fully connected layers, making it robust to unseen fungal images.

SOM was trained on the dataset to map input data points into a 2D grid of neurons. Each node represents a prototype vector summarizing the data characteristics of its neighborhood. During training, SOM organized similar data points into nearby nodes, forming clusters on the grid. After training, each neuron was assigned a label based on the majority class of the data points it represents. New data points were classified by finding the Best Matching Unit (BMU) on the SOM grid, which is the neuron closest to the input data in terms of Euclidean distance. The BMU’s label was then assigned to the input data point. The whole process for SOM is given below (1)–(7):

(1) $$X=\left\{x_{1},x_{2},x_{3},\ldots ,x_{N}\right\},\;x_{i}\in R^{d}$$

where X, N, and d represent the input dataset, the number of samples, and the dimensionality of each sample, respectively. Each neuron has a weight vector according to the grid size (2):

(2) $$w_{ij}\epsilon R^{d},\;i,j\;\in \left[1,\;20\right]$$

During the training phase, for each input x∈X, the BMU is found (3):

(3) $$BMU=\arg\underset{\mathrm{ij}}{\min}\left\|x-w_{ij}\right\|$$

where $\left\|\cdot \right\|$ is the Euclidean distance. The weights of the BMU and its neighbors are updated (4):

(4) $$w_{ij}\left(t+1\right)=w_{ij}\left(t\right)+\alpha \left(t\right)h_{BMU,ij}\left(t\right)\left(x-w_{ij}\left(t\right)\right)$$

where $\alpha \left(t\right)$ and $h_{BMU,ij}\left(t\right)$ represent learning rate and Gaussian neighborhood function, respectively. $h_{BMU,ij}\left(t\right)$ defines how much the neighboring neurons of the Best Matching Unit (BMU) are updated during training. It is given by (5):

(5) $$h_{BMU,ij}\left(t\right)=exp\left(-\frac{{\left\|r_{BMU}-r_{ij}\right\|}^{2}}{2\sigma {\left(t\right)}^{2}}\right)$$

where $r_{BMU}$ and $r_{ij}$ represent the coordinates of the BMU and the coordinates of the neuron in the SOM grid, respectively. $\left\|r_{BMU}-r_{ij}\right\|$ is the Euclidean distance between the BMU and the neuron $\left(i,j\right)$ in the grid. It is computed as (6):

(6) $$\left\|r_{BMU}-r_{ij}\right\|=\sqrt{{\left(x_{BMU}-x_{ij}\right)}^{2}+{\left(y_{BMU}-y_{ij}\right)}^{2}}$$

where $\sigma \left(t\right)$ represents the neighborhood radius at time $\left(t\right)$. A formula for $\sigma \left(t\right)$ can be given as (7):

(7) $$\sigma \left(t\right)=\sigma_{0}exp\left(-\frac{t}{\tau }\right)$$

where $\sigma_{0}$ and τ represent the initial neighborhood radius and time constant controlling the rate of decay, respectively. t stands for the current training iteration.

In the classification phase, each node in the grid is assigned a label based on the majority class of the input samples mapped to it. For a new input $x_{new}$, the BMU is found, and its label is used as the class prediction. For each input, in our case, class labels are represented as $y\in \left\{1,\;2,\;3,\;\ldots ,7\right\}$.

Additionally, SOM was also utilized to classify the features extracted from MaxViT-S. The features for each image were extracted by removing the final classification layer of the MaxViT-S model. In this scenario, the parameters, which are grid size, sigma, learning rate, and the number of iterations, were optimized to 10 × 10, 1.0, 0.5, and 1000, respectively.

A new method, an ensemble method (MaxViT-ResNet), was also implemented to classify fungi images. The final layers of both models are removed, and they are utilized solely for feature extraction purposes. In this scenario, the input images are simultaneously fed into both the MaxViT-S and ResNetV2-50 models. Independent feature vectors are obtained from the feature extraction layers of MaxViT-S and ResNetV2-50. The feature vectors for MaxViT-S and ResNetV2-50 have dimensions of 768 and 2048, respectively. The feature vectors are combined to form a unified feature vector with a dimension of 2816. In the classification phase, the unified vector is passed through a fully connected block, the architecture of which is shown in Table 4.

Additionally, in this study, the proposed CNN architecture for extracting features from fungi images was integrated with a custom layer block called the KAN layer to enhance the performance of the CNN model (CNN-KAN). The KAN layer includes a sine function and an additional linear layer to enhance the model’s classification capability. Specifically, the sine function provides non-linear transformations based on the Kolmogorov–Arnold theorem. Here, the potential of the non-parametric transformation to enhance the model’s generalization capability has been taken into consideration. The Kolmogorov–Arnold theorem states that any multivariate continuous function (e.g., a classification problem) can be expressed as the sum of a series of univariate functions (8):

(8) $$f\left(x_{1},\;x_{2},x_{3},\ldots x_{n}\right)=\sum_{i=1}^{N}\phi_{i}\left(\sum_{j=1}^{n}\psi_{ij}\left(x_{j}\right)\right)$$

where $f\left(x_{1},\;x_{2},x_{3},\ldots x_{n}\right)$, $\phi_{i}$, and $\psi_{ij}$ represent the output of the classification, continuous activation function, and univariate transformation function, respectively. In our case, the output from the CNN is a 256-dimensional vector. A sine-based transformation is applied to each input, resulting in a 7-dimensional output vector. The sequence of operations (hidden layer, sinus activation function, and output layer) is provided below (9)–(11):

(9) $$z=W_{1}\cdot x+b_{1},\;W_{1}\in R^{128\times 256},{x\in R^{128},b}_{1}\epsilon R^{128},\;z\in R^{128}$$

(10) $$h=\sin\left(z\right)$$

(11) $$y=W_{2}\cdot h+b_{2},\;{\;W}_{2}\epsilon R^{7\times 128},b_{2}\epsilon R^{7}$$

## 3. Results

### 3.1. Evaluation Metrics

Seven evaluation metrics were utilized in this study to obtain the performances of the different models. These metrics are macro-accuracy (*Acc*), precision (*Pre*), sensitivity or recall (*Sn*), specificity (*Sp*), F_1_ score (*F*_1_), Matthews Correlation Coefficient (MCC), and Area Under Curve (AUC). All the metrics are calculated by macro-averaging for K=7 classes. The definition and formula of each evaluation metric is given below (12)–(18).

*Accuracy* measures the overall correctness of the model by comparing the number of correctly classified samples to the total number of samples (12):

(12) $$Acc=\frac{1}{K}\sum_{i=1}^{K}\frac{{TP}_{i}}{{TP}_{i}+{FP}_{i}+{FN}_{i}}$$

where ${TP}_{i}$ represents correctly predicted instances of class i or true positives for class i; ${FP}_{i}$ represents false positives for class i; and ${FN}_{i}$ represents false negatives for class i.

Precision, also known as Positive Predictive Value, measures the proportion of correct positive predictions for a given class (13):

(13) $${Pre}_{i}=\frac{{TP}_{i}}{{TP}_{i}+{FP}_{i}}$$

$$Macro\;Pre=\frac{1}{K}\sum_{i=1}^{K}{Pre}_{i}$$

where ${TP}_{i}$ and ${FP}_{i}$ represent the true positives for class i, and instances predicted as class i but actually belong to other classes (false positives), respectively.

Sensitivity, also known as recall or True Positive Rate, measures the proportion of actual positives that are correctly predicted (14):

(14) $${Sn}_{i}=\frac{{TP}_{i}}{{TP}_{i}+{FN}_{i}}$$

$$Macro\;Sn=\frac{1}{K}\sum_{i=1}^{K}{Sn}_{i}$$

where ${FN}_{i}$ represents the instances of class i predicted as another class or false negatives for class i.

Specificity measures the proportion of true negatives correctly identified for a given class (15):

(15) $${Sp}_{i}=\frac{{TN}_{i}}{{TN}_{i}+{FP}_{i}}$$

$$Macro\;Sp=\frac{1}{K}\sum_{i=1}^{K}{Sp}_{i}$$

where ${TN}_{i}$ represents true negatives for class i or instances not belonging to class i, and not predicted as class i.

F_1_ score is the harmonic mean of precision and sensitivity for a class (16):

(16) $$F_{1i}=2*\frac{{Pre}_{i}*{Sn}_{i}}{{Pre}_{i}+{Sn}_{i}}$$

$$Macro\;F_{1}=\frac{1}{K}\sum_{i=1}^{K}F_{1i}$$

*MCC* is a balanced metric that considers all the elements of the confusion matrix. For multi-class problems, *MCC* is computed as (17):

(17) $$MCC=\frac{\sum_{i=1}^{K}\sum_{j=1}^{K}\sum_{k=1}^{K}\left(C_{ii}*C_{jk}-C_{ij}*C_{ki}\right)}{\sqrt{\left(\sum_{i=1}^{K}R_{i}\right)\left(\sum_{i=1}^{K}C_{i}\right)\left(\sum_{i=1}^{K}T_{i}\right)\left(\sum_{i=1}^{K}P_{i}\right)}}$$

where C, $C_{ij}$, $R_{i}$, $C_{i}$, $T_{i}$, and $P_{i}$ represent the confusion matrix, element at row i, column j of the confusion matrix, total actual instances of class i, total predicted instances of class i, true positives for class i, and total predicted positives for all the classes, respectively.

The Area Under the Curve (AUC) is a performance metric that evaluates the ability of a classification model to distinguish between classes. For multi-class classification, the AUC is generalized from the binary case by aggregating the pairwise comparisons between classes or averaging the AUC values across all the classes. A commonly used approach is the One-vs-Rest (OvR) method, where the AUC is computed for each class against the rest of the classes, and the overall AUC is averaged. For each class k, the binary AUC is calculated by treating class k as the positive class and all the other classes as the negative class (18):

(18) $${AUC}_{k}=\frac{1}{N_{k}N_{\neg k}}\sum_{i\in Class\;k}\sum_{j\in Class\neg k}1({\hat{p}}_{k,i}>{\hat{p}}_{k,j})$$

where 1(·) is the indicator function, which equals 1 if the condition inside is true and 0 otherwise. $N_{k}$ and $N_{\neg k}$ represent the number of positive and negative samples, respectively. ${\hat{p}}_{k,i}$ stands for the predicted probability for class k for the i-th sample.

The chi-square test for statistical significance can be used to compare two confusion matrices in a multi-class classification problem to assess whether the observed differences in classification outcomes between the two models are statistically significant.

The chi-square test evaluates whether there is a significant association between two categorical variables or whether the observed differences between the matrices arise due to random chance. In our context, it can determine if the differences in predictions (e.g., true positives, false positives, etc.) across classes between the two models are significant. To apply the chi-square test, a contingency table summarizing both confusion matrices is created. In the contingency table, the rows and the columns correspond to the actual classes and the predictions made by model 1 and model 2.

The chi-square statistic is defined as (19):

(19) $$\chi^{2}=\sum_{i=1}^{R}\sum_{j=1}^{C}\frac{{\left(O_{ij}-E_{ij}\right)}^{2}}{E_{ij}}$$

where $O_{ij}$ represents the observed frequency; $E_{ij}$ represents the expected frequency; R represents the number of rows or classes; and C represents the number of columns or predicted outcomes. In order to find the *p*-value (p), $\chi^{2}$ statistic is compared to the chi-square distribution with the calculated degrees of freedom (20):

(20) $$Degrees\;of\;Freedom=\left(R-1\right)*(C-1)$$

If p<0.05, the null hypothesis, which implies that differences are due to chance, is rejected.

### 3.2. Empirical Results

With the addition of the SOM and KAN layer to the proposed CNN architecture for classification purposes, the accuracy metric increased from 0.529 to 0.863 and 0.763, respectively (Table 5). Performance comparisons with the other pretrained models across seven evaluation metrics are also presented in Table 5.

The highest values across all the metrics were achieved with MaxViT-S. However, an analysis based solely on individual evaluation metrics may not be sufficient to determine whether these results carry statistically significant information. Therefore, a chi-square test was applied to the confusion matrices of all the proposed and pretrained models, as shown in Table 6.

In Table 6, the arrows pointing to the left and upward represent the performance superiority of the corresponding model and indicate that this performance superiority is statistically significant. The *p*-value results written in bold indicate that rather than the superiority of the respective model, the statistical significance is in favor of the models proposed by us.

According to Table 6, CNN-SOM outperformed only GoogleNet, and this superiority is also statistically significant. However, despite CNN-SOM not surpassing EfficientNet-B0, EfficientNetV2-M, and VGG19 based on evaluation metrics, the superiority of these models is not statistically significant.

The proposed CNN-KAN model did not outperform any of the pretrained models. However, the performance superiority of GoogleNet and VGG19 over CNN-KAN is not statistically significant.

MaxViT-SOM outperformed GoogleNet, MobileNet, EfficientNet-B0, EfficientNetV2, and VGG19. However, it performed worse than ResNetV2 and MaxViT-Small based on evaluation metrics. The superiority of MaxViT-SOM over GoogleNet is statistically significant, whereas its superiority over MobileNet, EfficientNet-B0, EfficientNetV2, and VGG19 is not statistically significant. Similarly, although MaxViT-SOM performed worse than ResNetV2 and MaxViT-S in terms of performance, the p-value results indicate that these performance differences are not statistically significant.

The proposed ensemble model (MaxViT-Small—ResNetV2-50) outperformed GoogleNet, MobileNet, and VGG19 in terms of performance. However, the performance superiority of the proposed model is statistically significant only when compared to GoogleNet. The performance degradation of the ensemble model compared to ResNetV2-50, EfficientNet-B0, EfficientNetV2-M, and MaxViT-S is not statistically significant.

## 4. Discussion and Conclusions

This study makes multifaceted contributions to the in-depth investigation of macrofungal diversity and morphological features, establishing itself as a significant work in the fields of mycology and ecology. Focusing on the *Mycena* and *Marasmius* genera, this research highlights the crucial roles of these fungal species in ecological systems and their relationship with human life [43,44]. For instance, species like *Mycena crocata* and *Mycena epipterygia* support the organic matter cycle, contributing to carbon and nutrient cycling [45]. Similarly, the iconic ecological behaviors of *Marasmius rotula* illustrate the economic and ecological importance of these species for both natural systems and humans [46]. Additionally, the study emphasizes the biotechnological potential of these fungal species [47]. Their applicability in both agricultural and industrial fields opens up significant avenues for novel biotechnological solutions [48]. However, the sensitivity of these species to various environmental factors is a critical aspect to consider when developing conservation strategies [49]. In particular, the effects of global climate change pose a significant threat to the distribution and population dynamics of these fungal species [50].

This research highlights the significant potential of deep learning algorithms in mycology and fungal taxonomy. The traditionally time-consuming and expertise-intensive nature of identification methods has been surpassed in terms of speed and accuracy through deep learning technologies [51,52]. This study demonstrates the successful classification of seven distinct fungal species using prominent deep learning models such as GoogleNet, ResNetV2, and MaxViT-S. The MaxViT-S model, with an accuracy rate of 98.9%, stands out [53,54,55]. This success can be attributed to the model’s effective integration of local and global attention mechanisms and its rapid analysis of multidimensional image features [56]. On the other hand, lightweight architectures like MobileNetV3-L have proven to be viable alternatives for energy-efficient applications [57]. The study also demonstrates how the integration of deep learning models with environmental data contributes to a broader understanding of fungal diversity [58,59]. However, the reliance of deep learning algorithms on large datasets and the requirement for high computational power pose challenges to their broader adoption [60,61]. Nevertheless, it is evident that these technologies will lay the foundation for next-generation analytical methods in fungal biology [62].

The comparison of deep learning-based models with traditional image-processing techniques reveals the striking advantages and limitations of these technologies. Older models like GoogleNet, with simpler architectures, offer lower accuracy rates (81.1%) compared to innovative models like MaxViT-S, which achieve an accuracy of 98.9% [63]. This disparity stems from the advanced feature enhancement capabilities of models that employ multidimensional attention mechanisms. Furthermore, deep learning algorithms stand out for requiring less manual intervention and effectively integrating diverse data sources compared to traditional techniques [64,65,66]. However, their high computational requirements and the need for optimized infrastructure can limit their applicability in resource-constrained environments.

The SOM architecture, typically used in unsupervised learning, has been adapted to supervised learning in this study and utilized for the first time in fungal classification. The proposed CNN architecture, when combined with SOM and a specialized KAN layer for classification purposes, resulted in an improvement in evaluation metric values. Furthermore, although the proposed CNN-SOM and MaxViT-SOM architectures yielded slightly lower performance compared to some pretrained models, this difference was found to be statistically insignificant. This study highlights that comparisons based solely on evaluation metrics may not always yield accurate conclusions; therefore, it is essential to validate the obtained results through statistical testing. In this study, a single layer of the KAN architecture was utilized instead of the entire KAN. In future studies, the KAN architecture will be optimized and employed. Collecting datasets with a larger number of fungi species and testing the relevant models is expected to lead to more accurate results in fungal classification.

The number of samples in the dataset can be considered as a limitation of the study. The dataset comprises 1582 images across seven fungal species, which is a moderate-sized dataset for deep learning applications. While larger datasets are preferable, we implemented several strategies to improve model generalization:

1. Data Augmentation: Techniques such as random rotations, horizontal flips, and color jittering artificially expand the dataset and help the model learn invariances to image transformations.
2. Pretrained Models: By leveraging transfer learning from large-scale image datasets (e.g., ImageNet), our models benefited from prior knowledge, reducing overfitting.
3. Regularization Techniques: We employed dropout layers and weight decay to prevent overfitting and enhance generalization.
4. Statistical Validation: The chi-square test was applied to assess the reliability of classification results across different models.
5. Independent Test Set: We allocated 30% of the dataset for independent testing, ensuring performance metrics reflect real-world applicability.

The high accuracy (98.9% with MaxViT-S) and statistical validation confirm that the models effectively generalize unseen fungal images.

In this study, a deep learning-based framework was developed for the classification of *Mycena* and *Marasmius* species, demonstrating its significant contributions to fungal ecology and taxonomy. Specifically, various models, including convolutional neural networks (CNNs), self-organizing maps (SOMs), and Kolmogorov–Arnold Networks (KANs), were employed to analyze the visual characteristics of macrofungal species. The results indicate that these models exhibit high classification accuracy on complex visual data and outperform traditional classification methods in terms of both speed and accuracy.

These approaches can be effectively utilized in outdoor applications where environmental conditions vary, such as autonomous fungal detection robots. In this context, the study highlights the suitability of a model supported by various data processing and augmentation techniques, ensuring resilience to environmental variations in field applications. The model’s flexibility in handling variations in lighting conditions, angles, and image quality provides a significant advantage in processing real-world data.

The proposed methodology has broad applicability in fields such as sustainable agriculture, ecosystem management, natural resource conservation, and biodiversity monitoring. Given the crucial role of fungi in soil quality and plant development, these classification systems can facilitate the accurate identification of fungal species in agricultural activities and contribute to the development of appropriate biological solutions. Moreover, fungi play a key role in the organic matter cycle, significantly impacting the carbon cycle and biodiversity. The accurate and efficient processing of such data can enhance the effectiveness of policies aimed at preserving ecological balance.

While expert mycologists and experienced mushroom pickers can often identify fungi based on visual features, accurate species-level identification remains challenging due to morphological similarities and variations caused by environmental factors. The proposed deep learning model offers several practical applications:

- Biodiversity Conservation: Automated identification can assist conservationists in mapping fungal distributions and tracking species at risk due to climate change or habitat destruction.
- Agriculture and Forestry: Some fungi are beneficial for soil health, while others are harmful pathogens. Accurate identification supports sustainable agriculture and forest management.
- Citizen Science and Education: Mobile applications utilizing our model can help amateur mushroom pickers and nature enthusiasts correctly identify species, reducing the risk of misidentification, especially for toxic mushrooms.
- Biotechnological Applications: Certain fungi have medicinal and industrial uses. Rapid classification aids in screening potential species for pharmaceutical and environmental applications.
- Autonomous Detection Systems: The model can be integrated into robotic or drone-based systems for real-time fungal identification in forests and agricultural fields.

This study demonstrates how deep learning can enhance species recognition beyond expert-level knowledge, making fungal identification more accessible and reliable.

This is the first study to integrate a supervised self-organizing map (SOM) with a CNN model for macrofungal classification. This method enhances feature clustering and interpretability. The manuscript provides a comprehensive comparison of CNN-SOM against state-of-the-art deep learning architectures, including MaxViT-S, ResNetV2-50, and EfficientNet models, using statistical validation methods. Unlike generic classification studies, our approach incorporates ecological and taxonomic considerations, making it more suitable for biodiversity research and conservation efforts. The proposed method can be implemented in autonomous fungal detection systems, mobile applications, and conservation monitoring tools, supporting mycologists and citizen scientists.

Furthermore, ecosystems affected by global climate change are undergoing significant shifts in fungal species distribution and population dynamics. This deep learning-based classification approach emerges as a valuable tool for monitoring environmental changes and analyzing their impact on fungal species. Future research will focus on testing these models with large datasets obtained from diverse geographical regions and ecosystems. In particular, real-time image acquisition and analysis through IoT-supported sensor networks in natural environments will enhance the system’s practical usability in the field. The development of such an integrated system will not only enable scientists to access field data more rapidly but also encourage public engagement in biodiversity studies. Additionally, integrating these systems with mobile applications and autonomous robots could lead to groundbreaking advancements in environmental monitoring efforts.

## Figures and Tables

**Table 1:** Information about the collected dataset.

Mushroom Species	Percentage of Images Received from Source	Number of Samples
*Marasmius oreades*	<22%	222
*Marasmius rotula*	<20%	228
*Mycena crocata*	<25%	229
*Mycena epipterygia*	<20%	243
*Mycena pura*	<23%	220
*Mycena rosea*	<25%	227
*Mycena seynii*	<25%	213

**Table 2:** The number of samples according to the training and test sets.

Mushroom Species	Training Set	Independent Test Set
*Marasmius oreades*	154	67
*Marasmius rotula*	159	69
*Mycena crocata*	160	69
*Mycena epipterygia*	171	72
*Mycena pura*	154	66
*Mycena rosea*	158	69
*Mycena seynii*	149	64

**Table 3:** The developed CNN architecture.

Name	Attributes
Convolutional Block 1	3 × 3 kernel size, 32 filters
Convolutional Block 2	3 × 3 kernel size, 64 filters
Convolutional Block 3	3 × 3 kernel size, 128 filters
Convolutional Block 4	3 × 3 kernel size, 256 filters
Global Average Pooling	
Fully Connected Layers	512 → 256 → 7 neurons
Dropout	50%

**Table 4:** The architecture of classification block for the ensemble method.

Layer	Attribute
Linear layer	Input: 2816 Output: 512
Activation	ReLU
Dropout	50% (to prevent overfitting)
Linear layer	Input: 512 (256 with dropout) Output: 7 (number of classes)

**Table 5:** Performance values for the proposed CNN models and different architectures.

Architecture	Accuracy	Precision	Recall	F1-Score	Specificity	MCC	AUC (OvR)
GoogleNet	0.811	0.811	0.812	0.809	0.969	0.780	0.968
MobileNetV3-L	0.899	0.899	0.899	0.899	0.983	0.882	0.987
ResNetV2-50	0.979	0.979	0.979	0.979	0.997	0.976	0.999
EfficientNet-B0	0.958	0.959	0.958	0.958	0.993	0.951	0.998
EfficientNetV2-M	0.964	0.964	0.964	0.964	0.994	0.958	0.998
VGG19	0.876	0.877	0.876	0.876	0.979	0.856	0.987
MaxVit-S	0.989	0.989	0.989	0.989	0.998	0.988	0.999
CNN	0.529	0.533	0.529	0.529	0.922	0.451	0.502
CNN-SOM	0.863	0.885	0.862	0.864	0.977	0.843	0.911
CNN-KAN	0.763	0.762	0.763	0.759	0.961	0.724	0.861
MaxVit-SOM	0.977	0.977	0.977	0.977	0.996	0.973	0.986
Ensemble (MaxVit-Small—ResNetV2-50)	0.935	0.936	0.935	0.935	0.989	0.925	0.96

**Table 6:** Chi-square test results (*p*-values).

Models	GoogleNet	MobileNetV3-L	ResNetV2-50	EfficientNet-B0	EfficientNetV2-M	VGG19	MaxVit-S
CNN-SOM	5.147 × 10^−4^ ←	2.167 × 10^−2^ ↑	6.107 × 10^−3^ ↑	1.305 × 10^−1^	5.898 × 10^−2^	1.066 × 10^−1^	1.850 × 10^−2^ ↑
CNN-KAN	2.195 × 10^−1^	9.431 × 10^−3^ ↑	4.893 × 10^−8^ ↑	9.977 × 10^−6^ ↑	3.629 × 10^−6^ ↑	1.411 × 10^−1^	1.332 × 10^−8^ ↑
MaxVit-SOM	1.217 × 10^−3^ ←	8.919 × 10^−1^	1.000	9.999 × 10^−1^	1.000	4.012 × 10^−1^	1.000
Ensemble MaxVit-ResNet	2.299 × 10^−2^ ←	6.475 × 10^−1^	9.999 × 10^−1^	9.588 × 10^−1^	1.000	6.569 × 10^−1^	9.874 × 10^−1^

## Data Availability

The data presented in this study are openly available in [Google Drive] at [https://tinyurl.com/wp8wefy8] (accessed on 4 March 2025), reference number [16].

## References

[1] Souto M., Raposeiro P.M., Balibrea A., Gonçalves V. (2024). Checklist of Basidiomycota and New Records from the Azores Archipelago. Diversity.

[2] Akata I., Kumru E., Ediş G., Özbey B.G., Şahin E. (2023). Three New Records for Turkish Agaricales Inhabiting Ankara University Beşevler 10th Year Campus Area. Kastamonu Univ. J. For. Fac..

[3] Antonín V., Noordeloos M.E. (2010). A Monograph of Marasmioid and Collybioid Fungi in Europe.

[4] Robich G. (2003). Mycena d’Europa.

[5] Knudsen H., Vesterholt J. (2008). Funga Nordica: Agaricoid, Boletoid, and Cyphelloid Genera.

[6] Aronsen A., Læssøe T. (2016). The Genus Mycena s.l. The Fungi of Northern Europe.

[7] Breitenbach J., Kränzlin F. (1991). Fungi of Switzerland, Volume 3: Boletes and Agarics 1. Part.

[8] Maas Geesteranus R. (1986). Conspectus of the Mycenas of the Northern Hemisphere—8. Sections Intermediae, Rubromarginatae. Proc. K. Ned. Akad. Wet. C Biol. Med. Sci..

[9] Asif M., Maula F., Saba M., Akram W., Raza M. (2024). Taxonomic and Phylogenetic Evidence Reveal a New Species and a New Record of the Genus *Marasmius* from Pakistan. Phytotaxa.

[10] Ozsari S., Kumru E., Ekinci F., Akata I., Guzel M.S., Acici K., Asuroglu T. (2024). Deep Learning-Based Classification of Macrofungi: Comparative Analysis of Advanced Models for Accurate Fungi Identification. Sensors.

[11] Yan Z., Liu H., Li J., Wang Y. (2023). Application of Identification and Evaluation Techniques for Edible Mushrooms: A Review. Crit. Rev. Anal. Chem..

[12] Picek L., Šulc M., Matas J., Heilmann-Clausen J., Jeppesen T.S., Lind E. (2022). Automatic Fungi Recognition: Deep Learning Meets Mycology. Sensors.

[13] Chathurika K., Siriwardena E., Bandara H., Perera G., Dilshanka K. (2023). Developing an Identification System for Different Types of Edible Mushrooms in Sri Lanka Using Machine Learning and Image Processing. Int. J. Eng. Manag. Res..

[14] Bartlett P., Eberhardt U., Schütz N., Beker H.J. (2022). Species Determination Using AI Machine-Learning Algorithms: *Hebeloma* as a Case Study. IMA Fungus.

[15] Global Core Biodata Resource. www.gbif.org.

[16] Macrofungi Species Dataset. https://tinyurl.com/wp8wefy8.

[17] Kohonen T. (1990). The Self-Organizing Map. Proc. IEEE.

[18] Sarker I.H. (2021). Deep Learning: A Comprehensive Overview on Techniques, Taxonomy, Applications and Research Directions. SN Comput. Sci..

[19] Kawaguchi T., Ono K., Hikawa H. (2024). Electroencephalogram-Based Facial Gesture Recognition Using Self-Organizing Map. Sensors.

[20] Gholami V., Khaleghi M.R., Pirasteh S., Booij M.J. (2022). Comparison of Self-Organizing Map, Artificial Neural Network, and Co-Active Neuro-Fuzzy Inference System Methods in Simulating Groundwater Quality: Geospatial Artificial Intelligence. Water Resour. Manag..

[21] Liu Z., Wang Y., Vaidya S., Ruehle F., Halverson J., Soljacic M., Hou T.Y., Tegmark M. (2024). KAN: Kolmogorov-Arnold Networks. arXiv.

[22] Firsov N., Myasnikov E., Lobanov V., Khabibullin R., Kazanskiy N., Khonina S., Butt M.A., Nikonorov A. (2024). HyperKAN: Kolmogorov–Arnold Networks Make Hyperspectral Image Classifiers Smarter. Sensors.

[23] Livieris I.E. (2024). C-KAN: A New Approach for Integrating Convolutional Layers with Kolmogorov–Arnold Networks for Time-Series Forecasting. Mathematics.

[24] Hollósi J., Ballagi Á., Kovács G., Fischer S., Nagy V. (2024). Detection of Bus Driver Mobile Phone Usage Using Kolmogorov-Arnold Networks. Computers.

[25] Hollósi J. (2024). Efficiency Analysis of Kolmogorov-Arnold Networks for Visual Data Processing. Eng. Proc..

[26] Ibrahum A.D.M., Shang Z., Hong J.-E. (2024). How Resilient Are Kolmogorov–Arnold Networks in Classification Tasks? A Robustness Investigation. Appl. Sci..

[27] Szegedy C., Liu W., Jia Y., Sermanet P., Reed S., Anguelov D., Erhan D., Vanhoucke V., Rabinovich A. Going Deeper with Convolutions. Proceedings of the IEEE Conference on Computer Vision and Pattern Recognition (CVPR).

[28] Singh V., Baral A., Kumar R., Tummala S., Noori M., Yadav S.V., Kang S., Zhao W. (2024). A Hybrid Deep Learning Model for Enhanced Structural Damage Detection: Integrating ResNet50, GoogLeNet, and Attention Mechanisms. Sensors.

[29] Simonyan K., Zisserman A. (2014). Very Deep Convolutional Networks for Large-Scale Image Recognition. arXiv.

[30] Alshammari A. (2022). Construction of VGG16 Convolution Neural Network (VGG16_CNN) Classifier with NestNet-Based Segmentation Paradigm for Brain Metastasis Classification. Sensors.

[31] Howard A., Sandler M., Chu G., Chen L.-C., Chen B., Tan M., Wang W., Zhu Y., Pang R., Vasudevan V. Searching for MobileNetV3. Proceedings of the IEEE/CVF International Conference on Computer Vision.

[32] He K., Zhang X., Ren S., Sun J. Deep Residual Learning for Image Recognition. Proceedings of the IEEE Conference on Computer Vision and Pattern Recognition.

[33] Li Z., Tian X., Liu X., Liu Y., Shi X. (2022). A Two-Stage Industrial Defect Detection Framework Based on Improved-YOLOv5 and Optimized-Inception-ResNetV2 Models. Appl. Sci..

[34] Tan M., Le Q. EfficientNet: Rethinking Model Scaling for Convolutional Neural Networks. Proceedings of the International Conference on Machine Learning (PMLR 2019).

[35] Abd El-Ghany S., Mahmood M.A., Abd El-Aziz A.A. (2024). Adaptive Dynamic Learning Rate Optimization Technique for Colorectal Cancer Diagnosis Based on Histopathological Image Using EfficientNet-B0 Deep Learning Model. Electronics.

[36] Tan M., Le Q. EfficientNetV2: Smaller Models and Faster Training. Proceedings of the International Conference on Machine Learning (PMLR 2021).

[37] Huang M.-L., Liao Y.-C. (2023). Stacking Ensemble and ECA-EfficientNetV2 Convolutional Neural Networks on Classification of Multiple Chest Diseases Including COVID-19. Acad. Radiol..

[38] Tu Z., Talebi H., Zhang H., Yang F., Milanfar P., Bovik A., Li Y. (2022). MaxViT: Multi-Axis Vision Transformer. Proceedings of the Computer Vision–ECCV 2022: 17th European Conference.

[39] Pacal I. (2024). Enhancing Crop Productivity and Sustainability through Disease Identification in Maize Leaves: Exploiting a Large Dataset with an Advanced Vision Transformer Model. Expert Syst. Appl..

[40] Liu Z., Lin Y., Cao Y., Hu H., Wei Y., Zhang Z., Lin S., Guo B. Swin Transformer: Hierarchical Vision Transformer Using Shifted Windows. Proceedings of the IEEE/CVF International Conference on Computer Vision.

[41] Pacal I. (2024). MaxCerVixT: A Novel Lightweight Vision Transformer-Based Approach for Precise Cervical Cancer Detection. Knowl.-Based Syst..

[42] MiniSom Library. https://github.com/JustGlowing/minisom.

[43] Harder C.B., Hesling E., Botnen S.S., Lorberau K.E., Dima B., von Bonsdorff-Salminen T., Kauserud H. (2023). Mycena Species Can Be Opportunist-Generalist Plant Root Invaders. Environ. Microbiol..

[44] Niego A.G.T., Rapior S., Thongklang N., Raspé O., Hyde K.D., Mortimer P. (2023). Reviewing the Contributions of Macrofungi to Forest Ecosystem Processes and Services. Fungal Biol. Rev..

[45] Thoen E., Harder C.B., Kauserud H., Botnen S.S., Vik U., Taylor A.F., Skrede I. (2020). In Vitro Evidence of Root Colonization Suggests Ecological Versatility in the Genus Mycena. New Phytol..

[46] Koch R.A., Liu J., Brann M., Jumbam B., Siegel N., Aime M.C. (2020). Marasmioid Rhizomorphs in Bird Nests: Species Diversity, Functional Specificity, and New Species from the Tropics. Mycologia.

[47] Bonugli-Santos R.C., dos Santos Vasconcelos M.R., Passarini M.R., Vieira G.A., Lopes V.C., Mainardi P.H., Sette L.D. (2015). Marine-Derived Fungi: Diversity of Enzymes and Biotechnological Applications. Front. Microbiol..

[48] Rame R., Purwanto P., Sudarno S. (2023). Biotechnological Approaches in Utilizing Agro-Waste for Biofuel Production: An Extensive Review on Techniques and Challenges. Bioresour. Technol. Rep..

[49] Dahlberg A., Genney D.R., Heilmann-Clausen J. (2010). Developing a Comprehensive Strategy for Fungal Conservation in Europe: Current Status and Future Needs. Fungal Ecol..

[50] Nnadi N.E., Carter D.A. (2021). Climate Change and the Emergence of Fungal Pathogens. PLoS Pathog..

[51] Li D., Hegde S., Kumar A.S., Zacharias A., Mehta P., Mukthineni V., Acharya S. (2024). Towards Transforming Malaria Vector Surveillance Using VectorBrain: A Novel Convolutional Neural Network for Mosquito Species, Sex, and Abdomen Status Identifications. Sci. Rep..

[52] Khalil A.F., Rostam S. (2024). Machine Learning-Based Predictive Maintenance for Fault Detection in Rotating Machinery: A Case Study. Eng. Technol. Appl. Sci. Res..

[53] Picek L., Šulc M., Matas J., Jeppesen T.S., Heilmann-Clausen J., Læssøe T., Frøslev T. Danish Fungi 2020—Not Just Another Image Recognition Dataset. Proceedings of the IEEE/CVF Winter Conference on Applications of Computer Vision.

[54] Jasim M.A., Al-Tuwaijari J.M. Plant Leaf Diseases Detection and Classification Using Image Processing and Deep Learning Techniques. Proceedings of the 2020 International Conference on Computer Science and Software Engineering (CSASE).

[55] Shoaib M., Hussain T., Shah B., Ullah I., Shah S.M., Ali F., Park S.H. (2022). Deep Learning-Based Segmentation and Classification of Leaf Images for Detection of Tomato Plant Disease. Front. Plant Sci..

[56] Li X., Li M., Yan P., Li G., Jiang Y., Luo H., Yin S. (2023). Deep Learning Attention Mechanism in Medical Image Analysis: Basics and Beyonds. Int. J. Netw. Dyn. Intell..

[57] Papa L., Proietti Mattia G., Russo P., Amerini I., Beraldi R. (2023). Lightweight and Energy-Aware Monocular Depth Estimation Models for IoT Embedded Devices: Challenges and Performances in Terrestrial and Underwater Scenarios. Sensors.

[58] Hernández Medina R., Kutuzova S., Nielsen K.N., Johansen J., Hansen L.H., Nielsen M., Rasmussen S. (2022). Machine Learning and Deep Learning Applications in Microbiome Research. ISME Commun..

[59] Rathnayaka A.R., Tennakoon D.S., Jones G.E., Wanasinghe D.N., Bhat D.J., Priyashantha A.H., Karunarathna S.C. (2024). Significance of Precise Documentation of Hosts and Geospatial Data of Fungal Collections, with an Emphasis on Plant-Associated Fungi. N. Z. J. Bot..

[60] Pandey M., Fernandez M., Gentile F., Isayev O., Tropsha A., Stern A.C., Cherkasov A. (2022). The Transformational Role of GPU Computing and Deep Learning in Drug Discovery. Nat. Mach. Intell..

[61] Zhou L., Pan S., Wang J., Vasilakos A.V. (2017). Machine Learning on Big Data: Opportunities and Challenges. Neurocomputing.

[62] Edwards J.E., Forster R.J., Callaghan T.M., Dollhofer V., Dagar S.S., Cheng Y., Smidt H. (2017). PCR and Omics-Based Techniques to Study the Diversity, Ecology and Biology of Anaerobic Fungi: Insights, Challenges and Opportunities. Front. Microbiol..

[63] Pacal I. (2024). Improved Vision Transformer with Lion Optimizer for Lung Diseases Detection. Int. J. Eng. Res. Dev..

[64] Pouyanfar S., Sadiq S., Yan Y., Tian H., Tao Y., Reyes M.P., Iyengar S.S. (2018). A Survey on Deep Learning: Algorithms, Techniques, and Applications. ACM Comput. Surv. (CSUR).

[65] Archana R., Jeevaraj P.E. (2024). Deep Learning Models for Digital Image Processing: A Review. Artif. Intell. Rev..

[66] Esteva A., Robicquet A., Ramsundar B., Kuleshov V., DePristo M., Chou K., Dean J. (2019). A Guide to Deep Learning in Healthcare. Nat. Med..

